# A FACILITATED APPROACH TO SUSTAINMENT PLANNING FOR A RURAL GERIATRICS PROGRAM

**DOI:** 10.1093/geroni/igae098.0981

**Published:** 2024-12-31

**Authors:** Eileen Dryden, Jessica Riley, Lauren Moo, Meaghan Kennedy, Camilla Pimentel, William Hung

**Affiliations:** Veterans Health Administration, Bedford, Massachusetts, United States; Veteran’s Affairs, Bedford, Massachusetts, United States; Department of Veterans Affairs, Bedford, Massachusetts, United States; Veterans Affairs, Bedford, Massachusetts, United States; VA Bedford Healthcare System, Bedford, Massachusetts, United States; James J Peters VA Medical Center, James J Peters VA Medical Center, New York, United States

## Abstract

Continual adaptations enhance health care program sustainability amid ongoing health system changes, yet implementation research lacks descriptions of processes to facilitate needed adaptions, particularly for programs implemented in heterogenous settings We employed a three-phase participatory method to explore potential program sustainment strategies for a 19-site Veterans Health Administration program (GRECC Connect) that uses a hub-and-spoke model to expand rural access to geriatric specialty care through telemedicine. First, hub site clinicians and staff completed the Program Sustainment Assessment Tool (PSAT), a publicly available online tool. All sites then participated in a virtual retreat at which potential sustainment strategies, barriers and facilitators to each strategy, the capacity to enact each proposed strategy, and data needed to move forward in choosing a sustainment path were discussed. And finally, sites engaged in a participatory process with their local GRECC Connect teams to draft a site-specific sustainment plan. Proposed sustainment paths included integrating with local or regional clinical services, or mentoring other groups wanting to provide geriatrics telehealth specialty care. Sites varied in their perceived confidence to sustain program activities. Barriers and facilitators to sustainment strategies related to the current level of integration into and relationships with existing clinical services, existing relationships with local and regional VA leadership, knowledge of facility priorities, and perceived capacity to develop their program’s business case. This participatory approach enabled sites to plan the most feasible sustainment strategy for their own site, while using the support, knowledge, and experience of the national GRECC Connect team to do so.

